# Secondary and 2-Year Outcomes of a Sexual Assault Resistance Program for University Women

**DOI:** 10.1177/0361684317690119

**Published:** 2017-03-02

**Authors:** Charlene Y. Senn, Misha Eliasziw, Karen L. Hobden, Ian R. Newby-Clark, Paula C. Barata, H. Lorraine Radtke, Wilfreda E. Thurston

**Affiliations:** 1 Department of Psychology/Women’s and Gender Studies Program, University of Windsor, Windsor, Ontario, Canada; 2 Department of Public Health and Community Medicine, Tufts University, Boston, MA, USA; 3 Department of Psychology, University of Guelph, Guelph, Ontario, Canada; 4 Department of Psychology, University of Calgary, Calgary, Alberta, Canada

**Keywords:** sexual assault, intervention, resistance, self-defense, randomized controlled trial

## Abstract

We report the secondary outcomes and longevity of efficacy from a randomized controlled trial that evaluated a novel sexual assault resistance program designed for first-year women university students. Participants (*N* = 893) were randomly assigned to receive the Enhanced Assess, Acknowledge, Act (EAAA) program or a selection of brochures (control). Perception of personal risk, self-defense self-efficacy, and rape myth acceptance was assessed at baseline; 1-week postintervention; and 6-, 12-, 18-, and 24-month postrandomization. Risk detection was assessed at 1 week, 6 months, and 12 months. Sexual assault experience and knowledge of effective resistance strategies were assessed at all follow-ups. The EAAA program produced significant increases in women’s perception of personal risk, self-defense self-efficacy, and knowledge of effective (forceful verbal and physical) resistance strategies; the program also produced decreases in general rape myth acceptance and woman blaming over the entire 24-month follow-up period. Risk detection was significantly improved for the intervention group at post-test. The program significantly reduced the risk of completed and attempted rape, attempted coercion, and nonconsensual sexual contact over the entire follow-up period, yielding reductions between 30% and 64% at 2 years. The EAAA program produces long-lasting changes in secondary outcomes and in the incidence of sexual assault experienced by women students. Universities can reduce the harm and the negative health consequences that young women experience as a result of campus sexual assault by implementing this program. *Online slides for instructors who want to use this article for teaching are available on PWQ’s website at http://journals.sagepub.com/page/pwq/suppl/index*.

The alarming rates of campus sexual assault were first documented nearly 30 years ago (in Canada: DeKeseredy & Kelly, 1993; in the United States: Koss, Gidycz, & Wisniewski, 1987), and these rates have not changed (e.g., Fisher, Cullen, & Turner, 2000; Krebs et al., 2016; Krebs, Lindquist, Warner, Fisher, & Martin, 2009). Male acquaintances perpetrate the vast majority of these attacks. Many women students will experience the negative physical and mental health effects of rape (e.g., Basile et al., 2006; Perilloux, Duntley, & Buss, 2012); the distal effects of increases in drug, alcohol, and tobacco consumption (e.g., Brener, McMahon, Warren, & Douglas, 1999; Deliramich & Gray, 2008; Young, Grey, Boyd, & McCabe, 2011); and the academic impacts such as lowered grades, dropping out of courses or university, and switching universities (Baker et al., 2016; Stermac, 2015). Any reduction in sexual assault will necessarily improve the health and well-being of young women on campuses.

Although there have been attempts to address the issue of sexual assault on North American campuses by changing perpetrator behavior, few interventions with men have been rigorously evaluated. Those that are effective usually show attitudinal changes for a few months, with no effect on the occurrence of rape or other forms of sexual assault; two recent exceptions combine bystander and social norms approaches (Gidycz, Orchowski, & Berkowitz, 2011; Salazar, Vivolo-Kantor, Hardin, & Berkowitz, 2014). The few other interventions shown to reduce perpetration when rigorously evaluated are for younger boys (for details, see reviews from Basile et al., 2016; DeGue et al., 2014). Currently, none of these programs is being widely implemented.

Yet, young women on campus continue to be confronted by sexual assault. Feminist self-defense or resistance (or risk reduction) education had promise in addressing this distressing reality (for reviews, see Brecklin, 2008; Gidycz & Dardis, 2014) when the first author began this research program. Although studies found consistent benefits of education programs in changing rape attitudes and women’s self-efficacy beliefs, the results were mixed with respect to reducing the sexual assaults women experienced. Most studies showed no impact for women with prior victimization, impact was observed for only a short time, and most interventions had no significant effects (Gidycz & Dardis, 2014).

The Enhanced Assess, Acknowledge, Act (EAAA) program was developed for first-year women university students to address limitations of the previous programs. It built on the strong foundation of prior feminist and feminist social psychological theory and research evidence. The EAAA program was piloted extensively and revised over 6 years (Senn, 2011; Senn, Gee, & Thake, 2011; Senn, Saunders, & Gee, 2008). In 2011, a multisite randomized controlled trial, named Sexual Assault Resistance Education (SARE), began (Senn et al., 2013). The EAAA program resulted in a 46% reduction in completed rape and 63% reduction in attempted rape, as well as significant reductions in other forms of sexual assault, compared to a control group (Senn et al., 2015). Further, the program was effective for women with and without a prior history of victimization. The trial positioned the EAAA program as the only intervention available for university campuses that provided Level 1 evidence for significantly reducing sexual assault among university women.

In brief, the EAAA sexual assault resistance program (named in tribute to the work of Rozee & Koss, 2001, who developed the Assess, Acknowledge, Act [AAA] name and concept) prepares women students for the statistical reality that a man they know may attempt to sexually assault them in a familiar social context (home, party, dorm). Built on the evidence-based cognitive ecological theory developed by Nurius and Norris (1996) to understand women’s cognitive, emotional, and behavioral responses at each stage of an acquaintance sexual assault, the EAAA program is designed to help women overcome emotional and cognitive barriers to detect and acknowledge the increased risk in men’s behavior. It also aims to assist women to more quickly take action using the most effective resistance strategies, particularly forceful verbal and physical tactics (e.g., Tark & Kleck, 2014; Ullman, 1997). Without such education, these effective strategies are the least likely to be used by women against men they know (Clay-Warner, 2002). The program makes clear that perpetrators are entirely responsible for the crimes they commit. It counteracts rape myths, specifically those that hold women responsible for men’s acts of sexual assault (Edwards, Turchik, Dardis, Reynolds, & Gidycz, 2011). For example, the content and activities directly address alcohol (presence of alcohol in a situation no matter who is using it) as one factor that can elevate risk of sexual assault; however, it is made clear that only the presence of a man willing to commit sexual assault generates any “risk” of sexual assault.

In addition to the primary trial outcomes related to sexual assault within the first year, the SARE trial collected data on five prespecified secondary outcomes specifically targeted by the EAAA program. It was hypothesized that the program would lead to (a) greater perception of personal risk of acquaintance rape; (b) earlier detection of risk in coercive situations; (c) higher confidence that one could defend oneself against sexual assault; (d) knowledge of (and willingness to use) more direct, forceful verbal and physical resistance strategies; and (e) decreased rape myth beliefs, including beliefs that women provoke rape through their actions. Because changes in attitudes, beliefs, and knowledge are likely to contribute to successful sexual assault resistance (Rozee & Koss, 2001) and better post-rape outcomes if a sexual assault occurs (Breitenbecher, 2006), analysis of these changes is important to ensure that the program content, as delivered, is affecting all theoretically important domains. In this article, we report the findings for all five prespecified secondary outcomes as well as the long-term efficacy of the primary sexual assault outcome. As no previous study has evaluated the efficacy of a sexual assault intervention beyond 1 year, the 2-year follow-up of participants afforded by the SARE trial has potential to inform whether the program requires boosters to retain its effectiveness in the long run.

## Method

The full protocol of this registered, open label, randomized controlled trial has been published elsewhere (Senn et al., 2013), as have the 12-month primary outcome results (Senn et al., 2015).

### Participants

**Figure 1.**
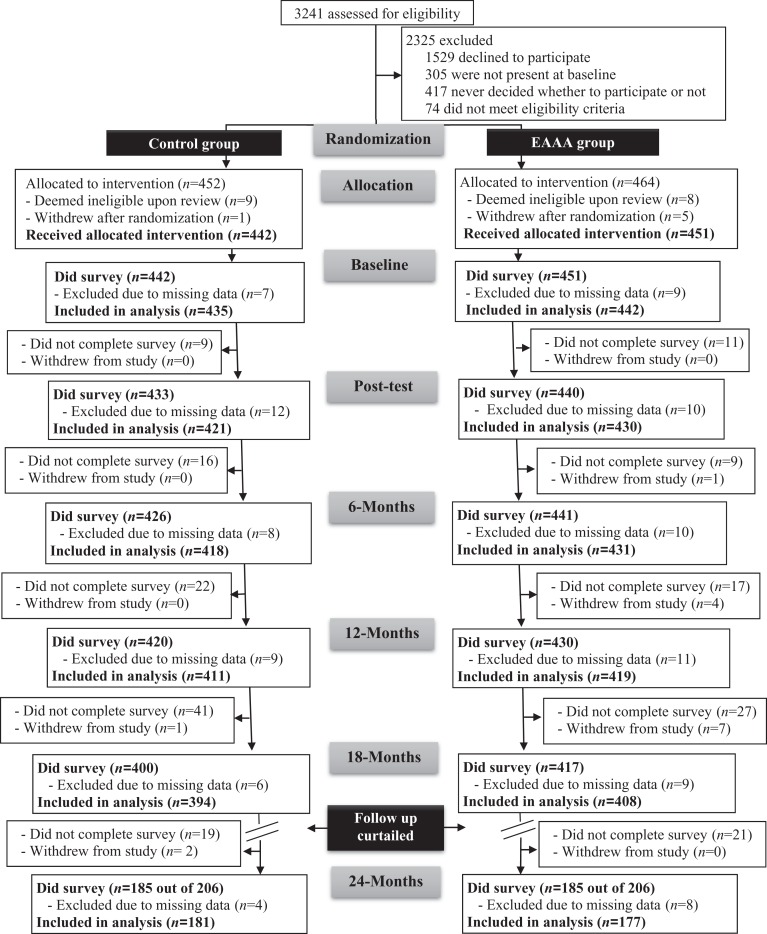
Flow diagram of progress through the phases of the Sexual Assault Resistance Education trial.

Over a 2-year period (2011–2013), 893 eligible first-year female undergraduate students from three Canadian universities, aged 17–24 years, were enrolled into the SARE trial. Of the 893 women who completed the baseline survey, 16 had missing data for one or more of the secondary outcome measures. Therefore, 877 were included in our analyses (Figure 1). Their average age was 18.5 years (*SD* = 1.2), almost three quarters were White (73.0%), most were heterosexual (91.8%), more than half were living in university residences (54.6%), and nearly one quarter had been previously raped (23.4%; see Senn et al., 2014, for additional baseline characteristics). The research was approved by institutional review boards at all three universities.

### Interventions

#### EAAA group

The EAAA program consisted of four 3-hr units designed for small groups (20 or fewer) of women led by two well-trained female facilitators. Each unit employed games, mini-lectures, facilitated discussions, small and large group exercises, as well as application and practice using written scenarios, audio and video clips, and role-play.

In Unit 1 (*Assess*), participants learned how to discern the level of risk for sexual assault present in situations involving male acquaintances and to develop problem-solving strategies aimed at minimizing the advantages of potential perpetrators. For example, the Assess unit identifies “presence of alcohol” (no matter who is drinking it) as one of the key situational cues that elevates risk of sexual assault. Activities in both *Assess* and *Acknowledge* units provide women with practice at identifying and undermining perpetrator advantages in situations that involve alcohol. This means that approximately 90 min of the 12 hr of EAAA programming includes discussion of the issue of alcohol-facilitated sexual assault, alone or in combination with other risk factors, and counteracts related myths. In Unit 2 (*Acknowledge*), the women were taught to recognize quickly the dangers inherent in situations that have turned coercive. They were provided with activities to help them develop strategies to prioritize their sexual rights and overcome the emotional barriers to seeing the danger and to engaging in resistance against men they know. In Unit 3 (*Act*), participants received instruction and practice on a variety of effective verbal and physical resistance strategies with a focus on common acquaintance sexual assault scenarios. In Unit 4 (*Relationships and Sexuality*), content from the previous three units was integrated and applied to participants’ sexual lives by providing a context for them to explore their own sexual and relationship values and desires as well as practice negotiating their needs (for more detail on the program content, see Senn et al., 2013, the supplementary appendix available on the website associated with the Senn et al., 2015, publication, or contact the first author).

Of the participants assigned to the EAAA group, all but two participants attended the first session (99%). Drop off in attendance was evident only between the first and second sessions; 89% attended each of the second, third, and fourth sessions. The average number of sessions attended by participants was 3.62 (*SD* = 0.82); 76% of participants attended all four sessions while 91% attended three or more sessions. Among the 17 previously reported participant characteristics (Senn et al., 2015), only 3 differed significantly in the percentage who attended three or four sessions. Participants were more likely to attend three or four sessions if they were not currently involved in a sexual relationship (94.4%) versus currently involved (86.1%), χ^2^(1, *N* = 451) = 8.96, *p* = .003; if they attended weekend sessions (95.1%) versus weekday sessions (88.1%), χ^2^(1, *N* = 451) = 6.14, *p* = .01; and if they had not been previously raped (92.8%) versus had a history of rape (83.5%), χ^2^(1, *N* = 451) = 8.18, *p* = .004.

#### Control group

Participants in the control group were given access to brochures pertaining to sexual assault and the opportunity to speak to someone knowledgeable about local sexual assault resources. The intention was to parallel the standard of care available to women students on university campuses at the time of data collection. The brochures used were specific to each of the three research sites, but their content was similar in covering general information on sexual assault and legal and medical advice for survivors/victims.

### Procedures

During the first (baseline) session, participants completed an in-person, computerized survey, were randomly assigned to either the EAAA group or control group using an online randomization tool (see Senn et al., 2015, for more detail), and then were sent to their appropriate intervention room. Participants were not informed of their intervention assignment until all participants had arrived at their assigned rooms. Highly trained facilitators used detailed manuals to deliver the interventions (EAAA group or control). Participants also completed an in-person, computerized survey at 1-week postintervention (post-test), with controls matched to the same time interval. Additional follow-up surveys were conducted online at 6, 12, 18 months, and, for half the participants, at 24 months. Funding did not allow follow-up of the entire cohort for 2 years. Participants were contacted up to 7 times at each follow-up time point to confirm receipt of the survey and to provide reminders. Incentives were offered for completing the baseline and postintervention surveys (e.g., bonus points in psychology courses, CDN$300 end of semester lottery) and for completing the follow-up surveys (CDN$30). Because of the greater time commitment required of participants assigned to the EAAA group, additional incentives were offered during each session (refreshments; small gifts such as My Body, My Choice whiteboard fridge magnets; CDN$50 session lottery).

Facilitators’ adherence to the session protocols (intervention fidelity) was assessed using checklists of content to be covered in each session. All sessions were audio recorded. For each semester, one quarter of the recordings for each primary facilitator was randomly selected for review by the trial project manager. Facilitators received a point for each item of content covered. The maximum number of content points varied by unit (Assess = 123, Acknowledge = 155, Act = 293, Relationships and Sexuality = 151). Intervention fidelity scores were converted to percentages for ease of comparison. The mean intervention fidelity score was 94% (range = 81–100% of content covered across facilitators and sessions).

### Outcome Measures

### Perceived risk of acquaintance rape

Participants rated on a 5-point scale (1 = *very unlikely*, 5 = *very likely*) “What are your chances of being raped by someone you know?” (adapted from Gray, Lesser, Quinn, & Brounds, 1990). Higher scores indicate greater perceived risk of acquaintance rape.

### Risk assessment

The two risk assessment measures, used at the post-test and at the follow-up, can be used only once. While they have different response scales, the construct they measure, assessment of a specific situation for risk of danger/negative outcomes as it unfolds, is similar.

Participants’ ability to detect risk was assessed at the post-test using a procedure and scale designed by Norris, Nurius, and Graham (1999) with additional items added by Testa, VanZile-Tamsen, and Livingston (2006). Participants read a scenario that described a woman on a date with an attractive male acquaintance (Michael). The man persists in physically touching the woman after she indicates she does not want him to (first coercion) and then uses his physical weight and force to assault her (second coercion). After each coercion segment, participants are asked, “How likely is it that the situation just described will result in…” and then to rate the probability of each of 10 possible outcomes (4 positive: e.g., “An evening that ends pleasantly?” and 6 negative: e.g., “You being upset by Michael’s behavior?”) on a 7-point scale (1 = *not at all likely*, 7 = *very likely*). The scores range from 10 to 70, with higher scores indicating higher risk of a negative outcome. Cronbach’s α was good (.81) in the current sample, but lower than that reported by Testa et al. (2006; α = .92).

Participants’ ability to detect risk also was assessed using scenarios and a procedure developed by Messman-Moore and Brown (2006). They were randomly assigned to receive one of the two versions (male acquaintance or male stranger) at 6 months and the other at 12 months. An interaction with a man is revealed online to participants line by line. After reading each line, participants indicate at which point they became uncomfortable (“Uncomfortable” line number) and at which point they would leave (“Leave” line number). The scores range from 1 to 25, with higher scores indicating tolerance of higher risk in the situation.

### Self-defense self-efficacy

Self-defense self-efficacy was assessed using Marx, Calhoun, Wilson, and Meyerson’s (2001) adaptation of Ozer and Bandura’s (1990) scale. Seven questions assessed women’s confidence, on a 7-point scale (1 = *not at all confident*, 7 = *very confident*), about their ability to defend themselves from men in a variety of situations (e.g., “If a man you were with was attempting to get you to have sex with him and you were not interested, how confident are you that you could successfully resist his advances?”). These were aggregated with 2 items written by the first author to more explicitly measure rape resistance: “How successful do you believe you would be in fighting off or otherwise stopping an attempted rape by a stranger [by a man you know (e.g., a man you are dating)]?” The expanded scores range from 9 to 63, with higher scores indicating greater self-defense self-efficacy or confidence that a woman could act in her own defense, and is reliable (current sample, α = .82; Senn et al., 2011, α = .83).

### Knowledge of effective rape resistance strategies

Two measures assessed participants’ knowledge of effective rape resistance strategies following the intervention. First, in the post-test survey only, participants read the two coercion segments used in the post-test measure of risk assessment described above (Norris et al., 1999) and rated the likelihood of various outcomes as described above. After each segment, they also indicated how likely they would be to engage in a number of responses. The analysis focused on the use of effective resistance strategies (the Direct subscale), which have a high level of reliability for both segments (current sample, α = .90; Norris et al., 2006, α = .85; Testa, VanZile-Tamsen, & Livingston, 2006, α = .96). The scores range from 6 to 42, with higher scores representing more direct (forceful) resistance. Second, at all postintervention time points, participants responded to the question “If a man I knew (e.g., a date or acquaintance) tried to force me to have sex with him when I didn’t want to, I would….” Using a coding system based on Ullman’s (1997) research on successful rape resistance strategies, these responses were scored for *whether* (1) or *not* (0) a participant mentioned an effective rape resistance strategy (i.e., forceful verbal or forceful physical response) and the number of instances of each strategy (e.g., punch, kick, and bite are all examples of forceful physical resistance). Cohen’s κs for interrater agreement were good to excellent, .82–.91 (Krippendorff, 1980; Landis & Koch, 1977). The number of instances ranged from 0 to 10, with higher scores indicating greater knowledge.

### Rape myth acceptance

The Illinois Rape Myth Acceptance Scale—Short Form (Payne, Lonsway, & Fitzgerald, 1999) consists of 17, seven-point items (1 = *not at all agree*, 7 = *very much agree*) that assess respondents’ belief in global rape myths (e.g., “Rape happens when a man’s sex drive gets out of control”). The scores range from 17 to 119, with lower scores indicating less subscription to rape myths and has very good reliability (current sample, α = .86; Payne et al., 1999, α = .93).

### Female precipitation of rape

The belief that women are responsible for rape was measured using the 6-item Female Precipitation subscale of the Perceived Causes of Rape Scale (Cowan & Campbell, 1995). The full scale was administered to maintain its integrity. Respondents were presented with the item “Rape is caused by…” and rated on a 7-point scale the extent to which they agreed with a list of causes (e.g., “…women who dress sexy”). The scores range from 6 to 42, with lower scores indicating less woman blaming, and has very good reliability (current sample, α = .86; Cowan & Campbell, 1995, α = .87).

### Sexual victimization

Participants’ experiences of sexual victimization were assessed using the Sexual Experiences Survey—Short Form Victimization (SES-SFV; Koss et al., 2007). The SES-SFV asks respondents to indicate the frequency of specific experiences that meet the legal definition for sexual assault (in Canada) and rape (in the United States). For example, “A man put his penis into my vagina, or inserted fingers or objects without my consent by…” “using force, for example holding me down with his body weight, pinning my arms, or having a weapon.” From these experiences, conventional scoring yielded five categories of sexual victimization: completed rape (oral, vaginal, or anal penetration by a man using threats, force, or drug/alcohol incapacitation), attempted rape, coercion (using pressure or manipulation to induce compliance), attempted coercion, and nonconsensual (non-penetrative) sexual contact. Each postintervention survey asked about the period of time since the last survey. Participants provided the date on which attempted and completed rapes occurred.

### Statistical Analyses

Linear (for comparing means) and generalized linear (for comparing proportions) mixed models were used to analyze the secondary outcomes data arising from the multilevel repeated-measures trial design. A random intercept was included in the models to account for the correlation among observations within group sessions, and a first-order autoregressive covariance structure was used to characterize the interdependence of the repeated measures over time. The models consisted of three terms: group (EAAA program or control), time (post-test, 6, 12, 18, or 24 months), and the cross-product between group and time. Results were summarized with corresponding 95% confidence intervals about group differences at each time point, and Cohen’s *d* was used to quantify the intervention effect sizes as “small, *d* = .2”; “medium, *d* = .5”; or “large, *d* = .8” (Cohen, 1988). A subset analysis, consisting of women assigned to the EAAA program, was also performed to assess whether the number of sessions attended (three or four vs. less than three) influenced the results. Incidences of completed and attempted rape were estimated from Kaplan–Meier failure analyses and compared between groups at each time point using Greenwood’s variance formula. Variances were inflated to account for within-session clustering. Incidences of coercion, attempted coercion, and nonconsensual sexual contact were estimated using actuarial life-table analyses, and the variances were also inflated. Results were summarized with corresponding 95% confidence intervals for group differences at each time point, and relative risk reductions were used to quantify intervention effect sizes. Log-rank tests were used to compare the groups over the entire follow-up period. All statistical analyses were conducted using SAS 9.3 (SAS Institute), and results with *p* values less than .05 were considered statistically significant.

## Results

Randomization yielded similar baseline characteristics between the two groups (Senn et al., 2015). At 18 months, 817 participants were included in the analyses, 400 in the control group and 417 in the EAAA group (Figure 1). Only participants enrolled in the first year of the trial’s recruitment period were invited to complete the 24-month survey, and therefore, only 370 participants (185 from each group) were included in the 24-month analyses.

### Effects of the EAAA Program

The EAAA program significantly increased participants’ perceptions of their personal risk of being raped by an acquaintance, *F*(1, 91) = 77.54, *p* < .001 (Table 1, row 1). The difference between the groups remained significant at each time point despite the effect sizes diminishing over time, from moderately large at the post-test (*d* = 0.71) to moderately small at 24 months (*d* = 0.37).

**Table 1.** Between-Group Comparisons of Perceived Risk of Acquaintance Rape, Self-Defense Self-Efficacy, Rape Myth Acceptance, and Belief in Female Precipitation of Rape Over Time.

			Baseline	Post-test	6 Months	12 Months	18 Months	24 Months	
			(*n* = 442)	(*n* = 430)	(*n* = 431)	(*n* = 419)	(*n* = 408)	(*n* = 177)	
Measure	Group		(*n* = 435)	(*n* = 421)	(*n* = 418)	(*n* = 411)	(*n* = 394)	(*n* = 181)	*p* Value^†^
Perceived risk of acquaintance rape	EAAA	Mean (*SE*)	1.84 (0.05)	3.45 (0.06)	3.07 (0.06)	3.11 (0.06)	3.17 (0.06)	3.10 (0.08)	<.001
	Control	Mean (*SE*)	1.81 (0.05)	2.52 (0.06)	2.38 (0.06)	2.55 (0.06)	2.59 (0.06)	2.68 (0.08)	.001
		Difference	0.03	0.93	0.69	0.56	0.58	0.42	
		95% CI	[−0.11, 0.17]	[0.76, 1.11]	[0.52, 0.87]	[0.39, 0.74]	[0.41, 0.77]	[0.18, 0.65]	
		Cohen’s *d*	0.03	0.71	0.53	0.44	0.45	0.37	
		*p* Value*	.66	<.001	<.001	<.001	<.001	<.001	
Self-defense self-efficacy	EAAA	Mean (*SE*)	44.0 (0.4)	53.6 (0.4)	51.4 (0.4)	51.2 (0.4)	51.1 (0.4)	51.3 (0.5)	<.001
	Control	Mean (*SE*)	44.9 (0.4)	47.2 (0.4)	47.1 (0.4)	47.7 (0.4)	47.7 (0.4)	47.9 (0.5)	.45
		Difference	−0.9	6.4	4.3	3.5	3.4	3.4	
		95% CI	[−2.0, 0.2]	[5.3, 7.5]	[3.2, 5.4]	[2.5, 4.7]	[2.3, 4.5]	[1.9, 4.8]	
		Cohen’s *d*	−0.11	0.80	0.54	0.45	0.43	0.49	
		*p* Value*	.11	<.001	<.001	<.001	<.001	<.001	
Rape myth acceptance	EAAA	Mean (*SE*)	32.1 (0.6)	23.7 (0.7)	25.4 (0.7)	25.3 (0.7)	25.1 (0.7)	24.8 (0.8)	.005
	Control	Mean (*SE*)	31.9 (0.6)	32.2 (0.7)	32.0 (0.7)	30.8 (0.7)	29.2 (0.7)	29.0 (0.8)	<.001
		Difference	0.2	−8.5	−6.6	−5.5	−4.1	−4.2	
		95% CI	[−1.6, 2.0]	[−10.4, −6.6]	[−8.5, −4.7]	[−7.4, −3.6]	[−6.1, −2.2]	[−6.6, −1.9]	
		Cohen’s *d*	0.02	−0.60	−0.47	−0.39	−0.30	−0.38	
		*p* Value*	.82	<.001	<.001	<.001	<.001	<.001	
Belief in female precipitation of rape	EAAA	Mean (*SE*)	15.2 (0.4)	10.0 (0.4)	10.9 (0.4)	10.6 (0.4)	10.5 (0.4)	10.2 (0.5)	.02
	Control	Mean (*SE*)	15.4 (0.4)	15.9 (0.4)	15.8 (0.4)	14.9 (0.4)	14.1 (0.4)	13.5 (0.5)	<.001
		Difference	−0.2	−5.9	−4.9	−4.3	−3.6	−3.3	
		95% CI	[−1.2, 0.8]	[−6.9, −4.8]	[−6.0, −3.8]	[−5.4, −3.2]	[−4.7, −2.5]	[−4.6, −1.9]	
		Cohen’s *d*	−0.02	−0.73	−0.61	−0.54	−0.45	−0.51	
		*p* Value*	.72	<.001	<.001	<.001	<.001	<.001	

*Note*. *SE* = standard error; CI = confidence interval; EAAA = Enhanced Assess, Acknowledge, Act program.

*p Value comparing means at each time point. ^†^*p* Value comparing postrandomization means.

The EAAA program also significantly increased participants’ risk assessment, *F*(1, 91) = 14.21, *p* < .001 (Table 2, row 1) as measured at the post-test by the Norris and colleagues’ scale (1999). As expected, respondents assessed their risk as being higher at the second instance of coercion than at the first, *F*(1, 91) = 1,076.00, *p* < .001.

**Table 2.** Between-Group Comparisons of Post-Intervention Measures of Risk Assessment and Use of Direct Resistance by Time of Coercion (First and Second).

			Time of Coercion		
			First Coercion	Second Coercion	
			(*n* = 430)	(*n* = 430)	
Measure	Group		(*n* = 421)	(*n* = 421)	*p* Value^†^
Risk assessment	EAAA	Mean (*SE*)	52.2 (0.5)	60.9 (0.5)	<.001
	Control	Mean (*SE*)	50.2 (0.5)	58.5 (0.5)	<.001
		Difference	2.0	2.4	
		95% CI	[0.7, 3.3]	[1.1, 3.7]	
		Cohen’s *d*	0.22	0.26	
		*p* Value*	.002	<.001	
Direct resistance	EAAA	Mean (*SE*)	28.1 (0.5)	36.3 (0.5)	<.001
	Control	Mean (*SE*)	24.0 (0.5)	32.4 (0.5)	<.001
		Difference	4.1	3.9	
		95% CI	[2.9, 5.4]	[2.6, 5.1]	
		Cohen’s *d*	0.44	0.41	
		*p* Value*	<.001	<.001	

*Note*. *SE* = standard error; CI = confidence interval; EAAA = Enhanced Assess, Acknowledge, Act program.

*p Value comparing means at each time of coercion. ^†^*p* Value comparing first and second coercion means.

There were no significant between-group differences on the follow-up measures of risk perception for acquaintances or strangers at 6 and 12 months (all *p*s > .20), and all effect sizes using Messman-Moore and Brown (2006) were tiny (all *d*s < .12). To allow for comparison with previous research, the results were summarized by aggregating the data across the two groups and across the two time periods, yielding: acquaintance (Uncomfortable, *M* = 8.20, *SD* = 3.83; Leave, *M* = 10.46, *SD* = 4.46; *N* = 710) and stranger (Uncomfortable, *M* = 12.70, *SD* = 6.72; Leave, *M* = 17.58, *SD* = 5.57; *N* = 731).

The EAAA program produced a sustained increase in women’s self-defense self-efficacy scores, *F*(1, 91) = 91.00, *p* < .001 (Table 1, row 2), and the difference between the groups remained significant at each time point despite the effect sizes diminishing over time, from large at the post-test (*d* = 0.80) to moderate at 24 months (*d* = 0.49).

The EAAA program produced a significant reduction in rape myth beliefs, *F*(1, 91) = 46.11, *p* < .001 (Table 1, row 3), and reductions remained significant at each time point despite the effect sizes diminishing over time, from moderately large at the post-test (*d* = −0.60) to moderately small at 24 months (*d* = −0.38).

The EAAA program significantly reduced the already low woman blaming beliefs held by the women, *F*(1, 91) = 83.97, *p* < .001 (Table 1, row 4), and reductions remained significant at each time point despite the effect sizes diminishing over time, from moderately large at the post-test (*d* = −0.73) to medium at 24 months (*d* = −0.51).

The EAAA program significantly increased participants’ effective (Direct) resistance strategies at the post-test, *F*(1, 91) = 44.41, *p* < .001 (Table 2, row 2). As expected, respondents indicated more direct resistance at the second instance of coercion than at the first, *F*(1, 91) = 1,249.64, *p* < .001. The EAAA program also significantly increased participants’ own generation of effective resistance strategies, both forceful verbal resistance, *F*(1, 874) = 40.24, *p* < .001; and forceful physical resistance, *F*(1, 874) = 138.62, *p* < .001; as well as more instances of forceful verbal resistance, *F*(1, 91) = 40.39, *p* < .001; and forceful physical resistance, *F*(1, 91) = 102.55, *p* < .001 (Table 3). The effect sizes ranged from moderately large (*d* = 0.61) to small (*d* = 0.19) and were sustained over time.

**Table 3.** Between-Group Comparisons of Mentioned Use of Effective Rape Resistance Strategies (Forceful Verbal and Forceful Physical) and Number of Effective Rape Resistance Strategies Suggested Over Time.

			Post-test	6 Months	12 Months	18 Months	24 Months	
			(*n* = 430)	(*n* = 431)	(*n* = 419)	(*n* = 408)	(*n* = 177)	
Measure	Group		(*n* = 421)	(*n* = 418)	(*n* = 411)	(*n* = 394)	(*n* = 181)	*p* Value^†^
Mentioned forceful verbal resistance	EAAA	Percentage (*SE*)	80.5 (1.9)	79.4 (1.9)	80.2 (1.9)	82.8 (1.9)	85.2 (2.6)	.47
	Control	Percentage (*SE*)	62.7 (2.4)	71.1 (2.2)	68.6 (2.3)	71.7 (2.3)	77.7 (3.0)	.001
		Difference (%)	17.8	8.3	11.6	11.0	7.5	
		95% CI	[11.9, 23.8]	[2.6, 14.1]	[5.7, 17.5]	[5.3, 16.8]	[−0.3, 15.4]	
		Cohen’s *d*	0.40	0.19	0.27	0.27	0.20	
		*p* Value*	<.001	.005	<.001	<.001	.06	
Number of instances of forceful verbal resistance	EAAA	Mean (*SE*)	1.26 (0.05)	1.21 (0.05)	1.24 (0.05)	1.32 (0.05)	1.28 (0.07)	.37
	Control	Mean (*SE*)	0.85 (0.05)	0.93 (0.05)	0.90 (0.05)	0.97 (0.05)	1.06 (0.07)	.05
		Difference	0.41	0.28	0.34	0.35	0.22	
		95% CI	[0.28, 0.55]	[0.14, 0.41]	[0.20, 0.47]	[0.21, 0.48]	[0.03, 0.41]	
		Cohen’s *d*	0.42	0.28	0.34	0.35	0.25	
		*p* Value*	<.001	<.001	<.001	<.001	.02	
Mentioned forceful physical resistance	EAAA	Percentage (*SE*)	76.8 (2.0)	69.4 (2.2)	70.2 (2.2)	68.3 (2.3)	71.8 (3.3)	.02
	Control	Percentage (*SE*)	44.4 (2.4)	49.1 (2.4)	43.9 (2.4)	47.6 (2.5)	45.6 (3.6)	.22
		Difference (%)	32.4	20.3	26.3	20.7	26.2	
		95% CI	[26.2, 38.6]	[13.8, 26.8]	[19.8, 32.8]	[14.1, 27.4]	[16.7, 35.7]	
		Cohen’s *d*	0.70	0.42	0.55	0.43	0.57	
		*p* Value*	<.001	<.001	<.001	<.001	<.001	
Number of instances of forceful physical resistance	EAAA	Mean (*SE*)	1.27 (0.06)	1.16 (0.06)	1.27 (0.06)	1.23 (0.06)	1.36 (0.09)	.13
	Control	Mean (*SE*)	0.60 (0.06)	0.70 (0.06)	0.64 (0.06)	0.74 (0.06)	0.66 (0.09)	.19
		Difference	0.67	0.46	0.63	0.49	0.70	
		95% CI	[0.51, 0.84]	[0.29, 0.62]	[0.46, 0.79]	[0.32, 0.66]	[0.46, 0.94]	
		Cohen’s *d*	0.55	0.37	0.51	0.40	0.61	
		*p* Value*	<.001	<.001	<.001	<.001	<.001	

*Note*. *SE* = standard error; CI = confidence interval; EAAA = Enhanced Assess, Acknowledge, Act program.

*p Value comparing means at each time point. ^†^*p* Value comparing postrandomization means.

**Figure 2.**
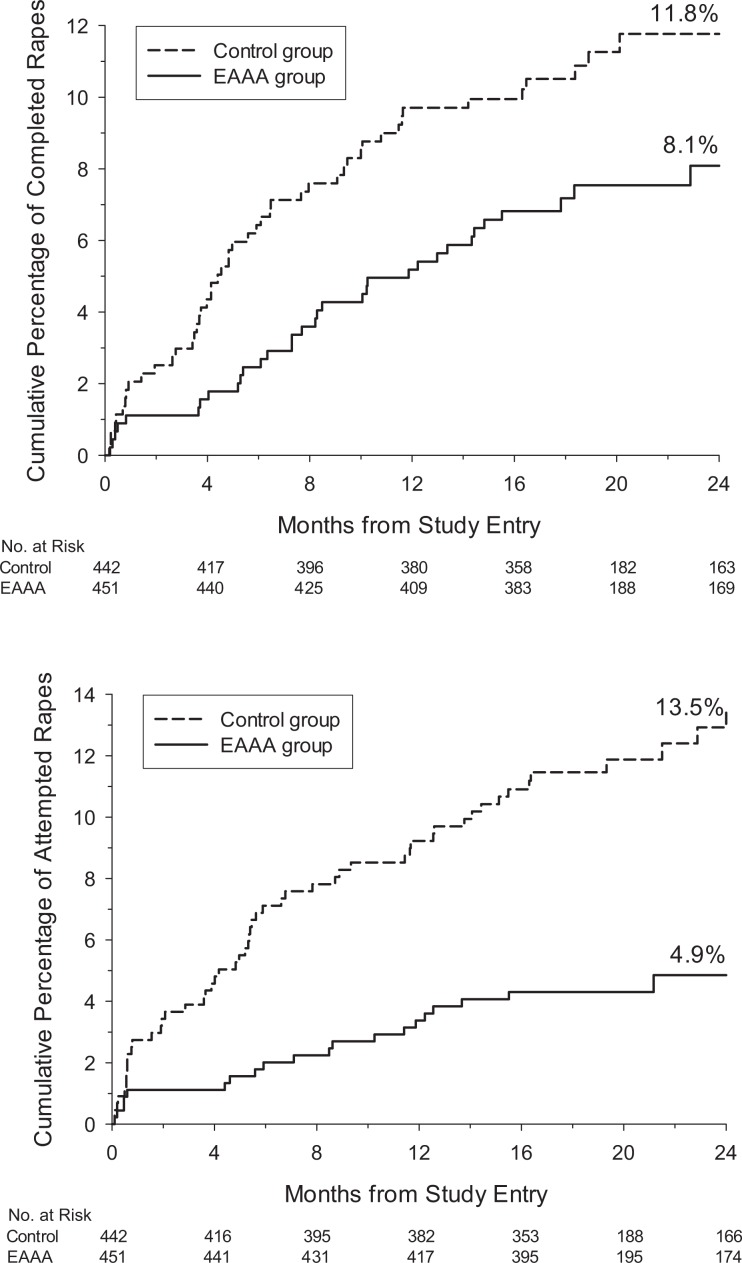
Top panel: Kaplan–Meier cumulative percentage of completed rapes over time. Bottom panel: Kaplan–Meier cumulative percentage of attempted rapes over time.

The EAAA program significantly reduced the risk (incidence) of completed rape as early as 6 months postintervention by 58.2%, *Z* = 2.35, *p* = .02, and continued to be efficacious up to 24 months, albeit with diminishing, no longer statistically significant, risk reductions (Table 4, row 1 and Figure 2, top panel). In contrast, the risk of attempted rape was significantly reduced over the entire 24-month follow-up period, *Z* = 3.98, *p* < .001, with sizable effects ranging from 55.8% to 71.8% (Table 4, row 2 and Figure 2, bottom panel). Although the risk of coercion was not significantly reduced at any time point, *Z* = 1.89, *p* = .06, the EAAA program consistently reduced the risk by one quarter. Finally, both attempted coercion and nonconsensual sexual contact were significantly reduced over the entire 24-month follow-up period, *Z* = 3.14, *p* = .002 and *Z* = 3.32, *p* = .001, respectively (Table 5, rows 2 and 3). In contrast to the other forms of sexual assault, effects on attempted coercion and nonconsensual sexual contact were detectable immediately at the post-test assessment, with sizable and significant reductions of 59.4% and 49.9%, respectively. However, similar to all other outcomes except attempted rape, the reductions diminished over time and toward the end of the study period leveled off at approximately 30%.

**Table 4.** Between-Group Comparisons of Completed Rape and Attempted Rape Over Time.

			Time Point				
			Post-test	6 Months	12 Months	18 Months	24 Months
			(*n* = 451)	(*n* = 445)	(*n* = 439)	(*n* = 424)	(*n* = 185)
Measure	Group		(*n* = 442)	(*n* = 434)	(*n* = 427)	(*n* = 412)	(*n* = 185)
Completed rape	EAAA	Percentage risk (*SE*)	1.1 (0.5)	2.7 (0.8)	5.2 (1.1)	7.2 (1.3)	8.1 (1.4)
	Control	Percentage risk (*SE*)	1.4 (0.6)	6.4 (1.2)	9.8 (1.4)	10.9 (1.5)	11.8 (1.6)
		Difference (%)	−0.3	−3.7	−4.6	−3.7	−3.7
		95% CI	[−1.9, 1.4]	[−6.9, −0.6]	[−8.5, −0.6]	[−8.1, 0.7]	[−8.5, 1.1]
		Relative reduction (%)	19.0	58.2	46.3	34.0	31.3
		*p* Value*	.76	.02	.02	.10	.13
Attempted rape	EAAA	Percentage risk (*SE*)	1.1 (0.5)	2.0 (0.7)	3.4 (0.9)	4.3 (1.0)	4.9 (1.1)
	Control	Percentage risk (*SE*)	2.5 (0.7)	7.1 (1.2)	9.3 (1.4)	11.9 (1.6)	13.5 (1.8)
		Difference (%)	−1.4	−5.1	−5.9	−7.6	−8.6
		95% CI	[−3.2, 0.4]	[−8.0, −2.3]	[−9.2, −2.5]	[−11.4, −3.8]	[−12.9, −4.3]
		Relative reduction (%)	55.8	71.8	63.2	63.8	63.9
		*p* Value*	.13	<.001	<.001	<.001	<.001

*Note*. *SE* = standard error; CI = confidence interval; EAAA = Enhanced Assess, Acknowledge, Act program.

*p Value comparing percentage risks at each time point.

**Table 5.** Between-Group Comparisons of Coercion, Attempted Coercion, and Nonconsensual Sexual Contact Over Time.

			Post-test	6 Months	12 Months	18 Months	24 Months
			(*n* = 451)	(*n* = 445)	(*n* = 439)	(*n* = 424)	(*n* = 185)
Measure	Group		(*n* = 442)	(*n* = 434)	(*n* = 427)	(*n* = 412)	(*n* = 185)
Coercion	EAAA	Percentage risk (*SE*)	2.4 (0.7)	8.0 (1.3)	10.7 (1.5)	12.8 (1.6)	13.1 (1.6)
	Control	Percentage risk (*SE*)	4.8 (1.0)	11.1 (1.5)	14.1 (1.7)	17.5 (1.8)	17.8 (1.8)
		Difference (%)	−2.4	−3.1	−3.4	−4.7	−4.7
		95% CI	[−4.8, 0.2]	[−7.1, 0.9]	[−7.9, 1.1]	[−9.6, 0.2]	[−9.7, 0.3]
		Relative reduction (%)	48.6	28.0	24.3	26.8	26.3
		*p* Value*	.07	.13	.13	.06	.06
Attempted coercion	EAAA	Percentage risk (*SE*)	3.8 (0.9)	10.2 (1.4)	15.0 (1.7)	17.3 (1.8)	17.6 (1.8)
	Control	Percentage risk (*SE*)	9.3 (1.4)	17.9 (1.8)	23.5 (2.0)	26.4 (2.1)	27.4 (2.2)
		Difference (%)	−5.5	−7.7	−8.5	−9.1	−9.8
		95% CI	[−9.1, −1.9]	[−12.8, −2.6]	[−14.3, −2.7]	[−15.1, −3.0]	[−15.9, −3.5]
		Relative reduction (%)	59.4	42.9	36.3	34.3	35.6
		*p* Value*	.003	.003	.004	.003	.002
Nonconsensual sexual contact	EAAA	Percentage risk (*SE*)	10.2 (1.4)	20.2 (1.9)	27.0 (2.1)	32.0 (2.2)	33.4 (2.3)
	Control	Percentage risk (*SE*)	20.4 (1.9)	33.6 (2.3)	42.0 (2.4)	45.5 (2.4	47.9 (2.4)
		Difference (%)	−10.2	−13.4	−15.0	−13.5	−14.5
		95% CI	[−16.7, −3.6]	[−21.4, −5.3]	[−23.6, −6.4]	[−22.4, −4.6]	[−23.6, −5.4]
		Relative reduction (%)	49.9	39.8	35.7	29.7	30.3
		*p* Value*	.002	.001	.001	.003	.002

*Note*. *SE* = standard error; CI = confidence interval; EAAA = Enhanced Assess, Acknowledge, Act program.

*p Value comparing percentage risks at each time point.

### Subset Analysis

In general, outcome effects were stronger among participants who attended more sessions (three or four) than fewer sessions (less than three). The following secondary outcomes reached statistical significance: Self-defense self-efficacy scores were significantly higher, *F*(1, 18) = 6.74, *p* = .02, *d* = 0.21; female precipitation of rape scores were significantly lower, *F*(1, 18) = 6.08, *p* = .02, *d* = −0.20; risk assessment scores were significantly higher, *F*(1, 16) = 8.41, *p* = .01, *d* = 0.36; and effective resistance strategies scores were significantly higher, *F*(1, 16) = 17.42, *p* < .001, *d* = 0.52. The stronger effects related to higher frequency of attendance were also mirrored by the pattern in the 24-month primary outcomes. Twenty-four-month incidences were lower (though these did not reach significance, likely due to small sample sizes) among participants who attended three or four versus less than three sessions: completed rape (7.4% vs. 15.4%, *Z* = 0.77, *p* = .44) and attempted rape (4.6% vs. 7.7%, *Z* = 0.48, *p* = .63).

## Discussion

The SARE trial evaluated the EAAA program. All five secondary outcomes related to the theoretical and empirical bases of the EAAA program content were significantly affected by the intervention. Thus, the EAAA program has long-lasting, positive effects on university women’s perceptions, attitudes and beliefs, and knowledge related to women’s ability to resist sexual assault by known men. Analysis of the trial’s sexual assault outcomes shows that the EAAA program’s positive effects continue for at least 2 years. This confirms EAAA as the only intervention with a large and sustained impact on the levels of sexual assault women experience while in university. Nevertheless, the diminishing reductions over time for several forms of sexual assault suggest that a booster may be required in the second year of university to maintain the larger effects observed in the first year of the trial.

### Effects of the EAAA Program

The EAAA program raised women’s perceptions of their own risk of acquaintance rape. Women moved from viewing rape as “unlikely” or “very unlikely” to the middle of the scale (neither likely nor unlikely). This shift may reflect the increased personal relevance of resistance education. Our pilot research demonstrated that participation in the program increased perceptions of personal risk without elevating fear of stranger rape (Senn et al., 2011). Prevention efforts are more effective when participants see themselves as potentially benefiting from the knowledge presented (Janz & Becker, 1984), although optimism biases regarding rape may be particularly difficult to influence (Gidycz, McNamara, & Edwards, 2006). While a single-item measure such as the one we adapted from previous research is never ideal, the analysis reported here confirms the program’s success in increasing assessment of personal risk.

A factor related to the perception of personal risk is the perception of risk in specific situations. Theory and research suggest that slow risk detection is a barrier to successful sexual assault resistance; acquaintance rape contexts (e.g., social situations, alcohol use) are particularly susceptible to slower detection (e.g., Norris, Nurius, & Dimeff, 1996). As hypothesized, 1 week following the intervention, women in the EAAA program were better at detecting risk in a hypothetical acquaintance rape situation than women in the control group. This was the case even in the early stages of the coercion where the perpetrator had not yet used force. However, there were no significant differences between the groups for the measure of risk detection at 6 and 12 months, contrary to our pilot results (Senn et al., 2011). This is despite the dramatic reductions in attempted rapes for 2 full years, which suggests detection of risk at an early stage of social interactions may be a critical benefit of EAAA. A methodological artifact may have undermined the measurement of these treatment effects at follow-up. In the pilot study, the measure of sexual victimization (the SES-SFV) was administered after the risk detection measure near the end of the survey, whereas in the SARE trial, it was administered before the risk detection measure. Comparison of trial scores on the Messman-Moore and Brown (2006) measure with published means suggests the early administration of the sexual assault measure may have acted as a prime for the detection of risk of sexual assault for all participants. As such, we are unable to make conclusions about the duration of improved risk detection.

Self-efficacy, or confidence that one can engage in the behavior being advocated, is key to behavioral change (Bandura, 1977). One of the most important aspects of sexual assault resistance programs and feminist or empowerment self-defense is increasing women’s self-defense self-efficacy (e.g., Brecklin, 2008). The effect sizes (as noted in the Results section) show that the EAAA program had a substantial positive impact on women’s confidence that they could resist sexual coercion and sexual assault.

Forceful verbal and physical tactics are both related to decreased likelihood of rape, regardless of whether the perpetrator is a stranger or acquaintance (e.g., Tark & Kleck, 2014; Ullman, 1997). Unfortunately, women less commonly employ these strategies when faced with sexual assault from men they know, particularly when the men are intimates (e.g., Clay-Warner, 2002). Successful resistance education, therefore, must increase women’s knowledge of, and willingness to use, the most effective self-defense methods against men they know. The results reported here indicate that women who take the EAAA program develop a “tool box” of effective strategies they are willing to use. The impact is particularly strong for physical resistance, which less than 50% of the control group spontaneously suggested at every time period, compared to more than 68% of the EAAA group. The number of both types of strategies proposed by women who took the program was also higher across 2 years, suggesting internalization of the program’s message that women employ multiple escalating tactics until they are safe.

Although women hold more favorable attitudes toward rape victims and have fewer rape myth beliefs than do men (Edwards et al., 2011), it is critical that sexual assault resistance programs undermine woman blaming attitudes for two reasons. First, sexual assault interventions for women must not inadvertently suggest that women are responsible for sexual assault, and second, internalization of woman blaming can lead to self-blame, a common phenomenon following rape that contributes to poor outcomes (e.g., Breitenbecher, 2006). The EAAA program was successful in reducing already low rape myth beliefs and woman blaming attitudes.

The present study is the first to report the effects of a campus sexual assault intervention beyond 1 year and one of the very few reporting effects beyond 6 or 7 months (see Hollander, 2014; Moynihan et al., 2014, for exceptions). Examining the results carefully, a few observations can be made. First, after the normal decline in scores from 1-week post-intervention (when program content could be easily called to mind) to the 6-month follow-up assessment, the effectiveness of the program on perceptions, attitudes, beliefs, and knowledge was maintained. Effects remained remarkably consistent and were beyond changes due to maturation, historical, or testing effects that are often present in longitudinal studies of students. Second, the EAAA program produced significant reductions in the number of women who experienced sexual assault and these effects were maintained up to 2 years, despite diminishing effect sizes. The robust findings related to the secondary outcomes suggest that there are no specific deficits in knowledge, attitudes, or skills appearing in the second year postintervention.

One possible reason for the diminishing reductions over time, particularly for completed rapes, could be changes in the contexts or relationships to the perpetrator, as women enter second year of university. There is some evidence that boyfriends become the more likely perpetrators of sexual assault as women progress through university (Smith, White, & Holland, 2003), which could introduce other forms of intimate partner violence into the equation. It is possible that the 12-hr program cannot address these particular circumstances sufficiently. Nation and colleagues (2003) reviewed effective youth health prevention and promotion programs and suggested that boosters are usually necessary to reinforce skills and reduce declines in positive impacts. Future research could explore and compare the context of completed rapes women experience across the first 2 years to identify what content and skills would need to be included in a targeted booster.

Our results suggest, not surprisingly, that dose matters. Women who attended more sessions appeared to receive greater benefit. A related strength of the EAAA program is its acceptability to first-year women students. Although it is a long program (12 hours), more than three quarters of the participants attended all four sessions and fewer than 10% missed more than one session. Post-session evaluations and explanations for absence provided to facilitators and research assistants suggested that students enjoyed the program and, when they missed sessions, they did so because of school or work responsibilities or illness. On only a very few occasions across all sites, survivors realized that their experience was too recent and dropped out of the study after completing the surveys or after the first session of the program. Some of these women took EAAA the following semester or year.

### Limitations and Suggestions for Future Research

Researchers developing and evaluating bystander-type prevention programs have identified the dearth of high-quality outcome measures as an issue (e.g., Banyard, Moynihan, Cares, & Warner, 2014). Resistance researchers on the other hand have benefited from a longer history of measure development and higher quality measures in most domains (e.g., sexual victimization, self-defense self-efficacy, rape myths), but gaps remain. Our study was limited by the use of a single-item measure of perception of personal risk of acquaintance rape (Gray et al., 1990). Development of a high quality multiple-item measure of this construct would move the field forward. The study was also limited in its capacity to evaluate the impact of EAAA on sexual violence risk detection, which is an important element of the logic model of how resistance education is expected to work. Two creative measures of sexual violence risk assessment for hypothetical situations are available (Messman-Moore & Brown, 2006; Norris et al., 1999); however, there are currently only three scenarios available across these two measures. This prevented us from testing these effects beyond three time points. Moreover, it is possible that the measures developed by Messman-Moore and Brown (2006) were affected by priming. Future research should develop additional unique stranger and acquaintance scenarios for these scales to permit longitudinal research measurement of risk detection. We also recommend that measures of risk assessment be presented before sexual victimization measures in future studies.

A large proportion of the sexual assaults committed by men against women students involve situations where women are unable to consent due to incapacitation by alcohol (Krebs et al., 2009; Testa & Hoffman, 2012). The EAAA program identifies alcohol (no matter who is drinking it) as a risk factor for sexual assault and provides women with a number of activities within which they can develop strategies to undermine any advantages that alcohol provides men who would commit sexual violence. Full sample reductions in sexual assault across the time periods suggest that alcohol-involved sexual assaults are among those being reduced though we did not design the study to specifically evaluate this. A future study could be designed to target sexual assaults where alcohol is the primary perpetrator weapon and investigate specific effects of EAAA or whether enhancements to program content related to alcohol-facilitated sexual assault would be beneficial.

The study findings are likely generalizable to any North American university campus, particularly because our sample across three universities of different sizes and characteristics was more demographically diverse than the samples represented in most intervention research in this field (for an exception, see Krebs et al., 2016). We do not know whether EAAA would have similar effects for less privileged young women who do not attend university, although we have some preliminary data suggesting it may be appropriate for younger girls (Senn, 2013). As expected in research on sexuality (or violence), where the topic is declared from the beginning, rather than masked during recruitment (e.g., Dunne et al., 1997; Saunders, Fisher, Hewitt, & Clayton, 1985; Wolchik, Braver, & Jensen, 1985), volunteer biases mean that participants have somewhat higher rates of sexual victimization entering the study (Senn et al., 2014) than found in the general student population (Krebs et al., 2009). This limitation is unlikely to affect conclusions related to the impact on secondary outcomes since the EAAA’s effects on the primary outcome were not significantly different for those with and without victimization (Senn et al., 2015).

### Practice Implications

The characteristics that make the EAAA program effective, such as an intensive program following best practices with two facilitators and a small group experience, require higher levels of investment of universities’ time and money for the EAAA, compared to that required for the common (ineffective) brief, large group format or online offerings (Lonsway et al., 2009). While future research may identify ways to reduce the length of the program while maintaining its effectiveness, our pilot studies during the development phase suggested that shorter units with less practice time led to effects with limited duration. A dismantling or optimization study may be necessary to aid in this work. Universities considering adopting the EAAA program and thinking about limits on resources would be wise to take into account the size of the effects found here for the sexual assault and secondary outcomes and the length of time they were still present without using a booster.

Universities have recently been encouraged to put prevention programs with known effectiveness in place on their campuses (Ontario Women’s Directorate, 2013; White House Task Force to Protect Students From Sexual Assault, 2014) and the Centers for Disease Control has now recognized the EAAA as one such effective program (Basile et al., 2016). However, administrations may be under pressure to just “check the box” and do something that is easier or costs less; this may result in the use of ineffective solutions. In this context, the intensiveness of EAAA could create an obstacle to implementation, or restrict the number of times EAAA would be offered at an institution, reducing the number of women students who could be reached. In some settings, the early or recent adoption of bystander-type interventions might be perceived to preclude anything else. Engaged and committed faculty and staff, as well as parents and students, can influence the decisions universities make regarding appropriate prevention.

We have addressed some possible barriers to implementation by directing interested universities to a well-respected, nonprofit organization website (not affiliated with the researchers) where the evidence for EAAA, the resources needed, and possible sources of funding have been detailed (http://www.blueprintsprograms.com/). In addition, a nonprofit organization (SARECentre.org) supports staff in their efforts to implement EAAA at their institutions. Our next study will examine the use of the EAAA program on a number of Canadian university campuses and investigate the campus, trainer, facilitator, and other factors related to effectiveness of the program outside of a randomized control study.

### Conclusions

The EAAA program is one critical piece of the solution for campus sexual assault. While we wait for broader social change through bystander education (e.g., Banyard, Eckstein, Plante, & Moynihan, 2007; Coker et al., 2011; Moynihan et al., 2014; Potter, Moynihan, Stapleton, & Banyard, 2009) and reductions in perpetration through widespread implementation of the few effective or promising interventions for boys (e.g., Foshee et al., 2004) and men (Gidycz et al., 2011; Salazar et al., 2014), implementation of the EAAA sexual assault resistance education program for female students is an important step universities can and must take now to reduce the harm and the negative health consequences that young women experience. The beneficial effects of the 12-hr EAAA program on reducing sexual assault are large. Offering the program with even limited reach would reduce the number of sexual assaults experienced by women in their first 2 years of university.

Some feminist scholars (e.g., Basile, 2008, as well as one of the reviewers for this article) have suggested that resistance education for women is misguided, in that perpetrators may simply move on to another woman. In other words, these critics suggest that sexual assault is prevented for the individual woman who resists, but that this would not necessarily reduce rates of sexual assault in the community overall. We, along with other feminist scholars (e.g., Hollander, 2016), suspect that perpetrators may learn important lessons that then have an impact on their subsequent behavior, when their intentions are detected early and their actions thwarted by bystanders or by the women they have targeted. We call for wide implementation to ensure that this message is repeated and amplified. Of course, which explanation is correct is an empirical question that could be tested in future research.

Based on our findings and experience, we recommend a joint call for comprehensive sexual assault prevention that combines the best of what the field currently has to offer. The best comprehensive strategy currently is education to influence the campus culture and teach students how to intervene on others’ behalf and, for the majority of sexual assault situations where there is no bystander present (Fisher et al., 2000), education to provide women with the knowledge and tools they need to intervene on their own behalf.
